# Estimation of Gestational Age via Image Analysis of Anterior Lens Capsule Vascularity in Preterm Infants: A Pilot Study

**DOI:** 10.3389/fped.2019.00043

**Published:** 2019-02-20

**Authors:** Monalisa Patel, Dibyendu Mukherjee, Sina Farsiu, Breda Munoz, Arlin B. Blood, Christopher G. Wilson, Jennifer B. Griffin

**Affiliations:** ^1^Department of Pediatrics, Loma Linda University, Loma Linda, CA, United States; ^2^Department of Biomedical Engineering, Duke University, Durham, NC, United States; ^3^Department of Ophthalmology, Duke University Medical Center, Durham, NC, United States; ^4^Applied Public Health Research Center, RTI International, Research Triangle Park, Durham, NC, United States; ^5^Lawrence D. Longo Center for Perinatal Biology, Loma Linda University School of Medicine, Loma Linda, CA, United States; ^6^Department of Ophthalmology, Loma Linda University, Loma Linda, CA, United States; ^7^Center for Global Health, RTI International, Research Triangle Park, Durham, NC, United States

**Keywords:** anterior lens capsule, lens vasculature, image analysis, preterm, gestational age, neonatal, smartphone ophthalmoscope

## Abstract

**Introduction:** Anterior lens capsule vascularity (ALCV) is resorbed in the developing fetus from 27 to 35 weeks gestation. In this pilot study, we evaluated the feasibility and validity of combining smartphone ophthalmoscope videos of ALCV and image analysis for gestational age estimation.

**Methods:** ALCV videos were captured longitudinally in preterm neonates from delivery using a PanOptic® Ophthalmoscope with an *iExaminer*® adapter (Welch-Allyn). ALCV video frames were manually selected and quantified using semi-automatic image analysis. A predictive model based on ALCV features was compared to gold-standard ultrasound gestational age estimates.

**Results:** A total of 64 image-capture sessions were carried out in 24 neonates. Ultrasound-estimated gestational age and ALCV-predicted gestational age estimates indicate that the two methods are similar (*r* = 0.78, *p* < 0.0001). ALCV estimates of gestational age were within 0.11 ± 1.3 weeks of ultrasound estimates. In the final model, gestational age was predicted within ± 1 week for 54% and within ± 2 weeks for 86% of the measures.

**Conclusions:** This novel application of smartphone ophthalmoscopy and ALCV image analysis may provide a safe, accurate and non-invasive technology to estimate postnatal gestational age, especially in low income countries where gestational age may not be known at birth.

## Introduction

While the past decade has seen significant reductions in under-five mortality, progress in reducing neonatal mortality has been slower. Currently, deaths during the neonatal period compose 41% of under-five deaths (1). The reasons for inadequate progress in reducing neonatal mortality are multifactorial; however, one critical issue is that neonates with the greatest risk of mortality are frequently not identified in low-income countries (LICs). Up to three-quarters of babies are not weighed at birth in sub-Saharan Africa and South Asia (2) and even fewer have a known gestational age (GA) at delivery (3). Accurate GA assessment in LICs is uncommon due to inaccuracies of last menstrual period dating, the complexity of neuro-physical assessments (e.g., Ballard), and the infrequency of prenatal ultrasound. Without accurate GA dating in LICs, at-risk babies may not receive simple, low-cost interventions that could save lives (4). Further, both epidemiological and programmatic research on premature birth and intrauterine growth restriction are hindered in LICs due to a lack of GA assessment. Novel approaches for assessing GA in LICs are critical.

The disappearance of anterior lens capsule vascularity (ALCV), a normal embryological process, has a high correlation with GA at delivery among preterm neonates from 27 to 35 weeks gestation (5). Examination of ALCV following delivery with a direct ophthalmoscope can be used to estimate GA. In 1977, Hittner et al. classified the disappearance of ALCV into four grades, from Grade 4, at 27 to 28 weeks gestation, through Grade 1, at 34 to 35 weeks gestation (5). Assessing ALCV may not be feasible prior to 27 weeks gestation because eyelids may be fused or the cornea may be too opaque to permit visualization. ALCV can typically be visualized up to 35 weeks of gestation, after which the vessels usually are resorbed completely (6).

To address the need for novel GA assessment methods in LICs, we conducted a pilot study to test the hypothesis that ALCV could be quantified with images captured via smartphone ophthalmoscopy and combined with semiautomated ALCV image analysis, providing a potentially clinically useful method to estimate GA in preterm neonates. We tested this hypothesis by collecting ALCV videos in preterm neonates; developing software for automated quantification of the ALCV features; and, by developing an algorithm to predict GA at delivery in preterm neonates.

## Materials and Methods

### Subject Selection

This prospective, non-randomized observational pilot study was approved by the Institutional Review Board at Loma Linda University, and written informed consent was obtained from parents or guardians of all subjects prior to enrollment. The study was performed in the Neonatal Intensive Care Unit of Loma Linda University Children's Hospital. Preterm infants were eligible for enrollment if they were < 48 h of age and born between 27 and 37 weeks gestation, based on early antenatal ultrasound (<20 weeks gestation). Infants were excluded from enrollment if they had fused eyelids or opaque corneas, were on high frequency oscillators, pressors, or had cardiorespiratory instability.

### Image Capture

ALCV was assessed in each infant at <48 h after birth and then every 5 days thereafter until no visible vascularity remained, 25 days after birth, or discharge, whichever occurred first. ALCV images were captured with either an iPhone 4 (5 megapixels) or iPhone 6s (12 megapixels) using MoviePro, a high-resolution video application (Deepak Sharma). The iPhone was attached to a PanOptic Ophthalmoscope with the iExaminer adapter (WelchAllyn, Skaneateles Falls, NY).

With the infant in the supine position, the ophthalmoscope was mounted on a ring stand and positioned with the camera lens parallel to and 4–5 cm above the surface of the eye. The phone, ophthalmoscope, and stand were disinfected with antiseptic wipes before and after each imaging session. The infant's temperature, heart rate, pulse-oximetry, and breathing were continuously monitored throughout the procedure and the examination was ceased in the event of bradycardia, desaturation, or apnea. Two drops of sterile saline were placed in the eye before imaging to prevent mucus artifacts. The upper and lower eyelids were then retracted using either gloved fingertips or cotton-tipped applicators until the entire pupil was visible. Between one and four videos <30 s duration were recorded at each imaging session. The imaging sessions took ~5 min. The examination was considered complete after a video with sufficient image quality was acquired.

### ALCV Quantification

The best frames from videos were manually selected based on optimal visualization of the pupil and focus of vasculature. ALCV features were then computed via semiautomated image analysis, the steps of which included: automatic extraction of the pupil from the selected image, color enhancement, segmentation, and morphological analysis of visible vasculature. The automatic extraction of the pupil consisted of the following steps: (i) extraction of all circular and elliptical objects from the image using a circular Hough transform (7); (ii) selection of the best candidate pupil based on weighted voting of contrast difference, amount of circularity, and color; and, (iii) segmentation of the pupil using information from both RGB (red-green-blue) and HSV (hue-saturation-value) color domains for better boundary extraction (8). Prior to segmentation, a color enhancement step was carried out using a contrast stretching algorithm on the red and green color channels to make the vessels more salient (7). The vessel segmentation followed a semi-automatic process where a hysteresis-based intensity thresholding algorithm automatically segmented the vasculature (7). The segmentation was verified and corrected via manual tuning of the feature extraction if required, before final quantification of ALCV features. The segmented vasculature formed a binary image. The final step of morphological analysis of the vasculature is as follows: (i) computation of the vessel skeleton to easily divide the vasculature map in short branches; (ii) measurement of the length, the width of each branch at each location on the branch, the average width, and the tortuosity of the branch using fractal dimension; and, (iii) computation of the imaging biomarkers: number of branches, largest and smallest branch length, width, and tortuosity, and the vasculature density. Where more than one video was taken at a single imaging session, the mean value for imaging biomarkers was used in the analysis. All image analysis and ALCV quantification was performed using MATLAB (9).

### Predictive Model Development

We developed a predictive model using R (10) that incorporated the parameters quantified using our automated detection software. We used a mixed linear model to account for the repeated ALCV measures and incorporated the following predictors: minimum and maximum branch length, minimum and maximum branch width, minimum and maximum tortuosity, density, and number of branches. We assessed the degree of association of each of these ALCV features with respect to GA as determined by ultrasound. Multiple models were investigated before developing a model that was optimized for multiple criteria [Minimum Variance Inflation Factor for Multicollinearity assessment (11), Akaike Information Criterion, Bayesian Information Criterion, and agreement between Bland-Altman, mean-squared deviation, and coverage probability]. We described the postnatal disappearance of ALCV statistically and graphically. Additionally, we tested agreement between ultrasound-determined and ALCV-determined GA using Bland-Altman plots with 95% limits of agreement (LOA) (12).

## Results

Of the 41 neonates screened, 13 were ineligible for enrollment due to lack of antenatal ultrasound <20 weeks GA (*n* = 3), fused eyelids (*n* = 3), or age >48 h at arrival to the Neonatal Intensive Care Unit (*n* = 7). Twenty-eight infants fulfilled the criteria for the study and were enrolled with longitudinal imaging. Four babies were withdrawn from the study early due to hemodynamic instability (*n* = 2) or parental refusal of subsequent imaging (*n* = 2). Of the 24 babies that underwent serial imaging, 21 had at least 3 serial imaging sessions. In total, 74 image capture sessions were completed. The mean ultrasound GA at the time of image capture was 33.2 weeks (range: 28.0–37.0; SD: 2.0).

**Figure 1:**
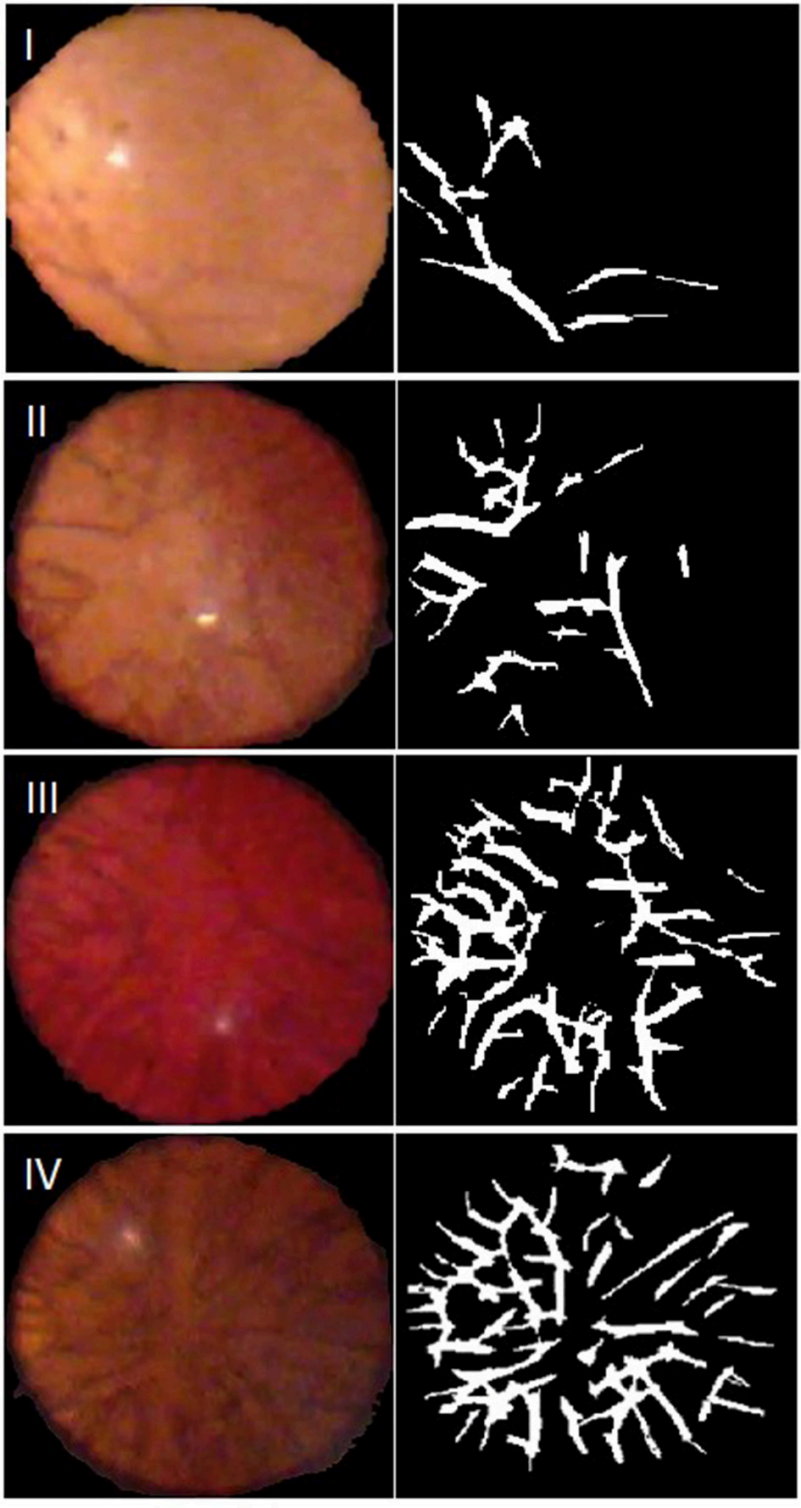
Representative video stills, with Hittner Grading, and their generated binary forms. Grade I: 33–34 weeks gestation; Grade II: 31–32 weeks gestation; Grade III: 29–30 weeks gestation; Grade IV: 27–28 weeks gestation.

Of the 74 video capture sessions completed, 64 sessions contained images that were deemed of sufficient quality for the automated ALCV quantification. Of the 10 excluded video sessions, 8 were completed on the iPhone 4 and two were completed on the iPhone 6 plus. All excluded videos were due to inadequate retroillumination of the lens. Representative examples of images captured for each of the four Hittner stages of ALCV and corresponding binary forms are shown (Figure 1).

**Figure 2:**
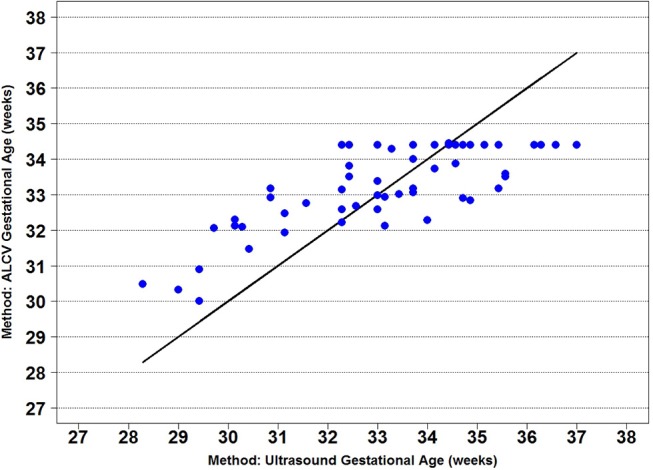
Identity plot of anterior lens capsule vascularity (ALCV) gestational age against ultrasound gestational age.

**Figure 3:**
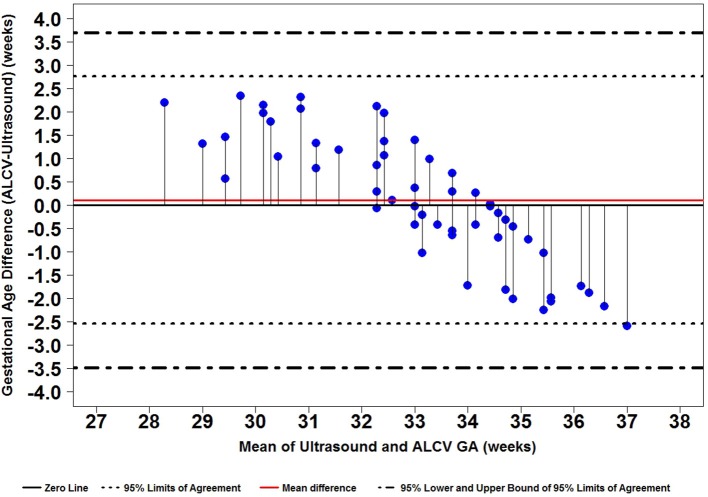
Bland-Altman plot of difference in gestational age against the mean of the two methods.

Seven parameters from the automated ALCV quantification were used to develop the GA predictive model (Table 1). The data points for ALCV GA and ultrasound GA fall adjacent to the identity plot line, suggesting some disagreement between the two methods (Figure 2). The correlation coefficient was 0.78 (*p* < 0.0001). One out of 64 data points are outliers (1.6%), exceeding the lower limit of agreement (Figure 3). The mean GA was similar for ALCV and ultrasound dating, with ALCV GA biased toward overestimation of 0.11 weeks (95% LOA: −2.6, 2.8). ALCV GA tended to overestimate GA in lower GA ranges and underestimate GA in higher GA ranges. The Bland Altman plot suggests that differences are greater between 35 and 37 weeks gestation than other gestational ages. The GA was predicted within ± 1 week for 48% of the measures and within ± 2 weeks for 86% of the ALCV GA estimates compared to ultrasound GA.

**Table 1:** Final ALCV model parameters for prediction of gestational age (*n* = 64).

**Parameter**	**Estimate**	**Standard error**	***p*-value**
Intercept	34.406	0.316	<0.0001
Branch length (max)	0.022	0.009	0.020
Branch width (max)	−0.092	0.077	0.241
Branch width (min)	0.155	0.228	0.502
Branch thickness (max)	−0.122	0.116	0.297
Branch thickness (min)	−1.667	0.650	0.015
Density	−0.124	0.050	0.019

## Discussion

In this pilot study, we found that smartphone ophthalmoscopy and semiautomated image analysis can quantify ALCV features and estimate GA among preterm neonates with a high level of agreement with ultrasound estimated gestational age. Postnatal GA estimation methods are useful in bedside estimation of GA, particularly in LICs where there is limited availability of ultrasound and other accurate GA dating methods. In this pilot study to develop methodology, ALCV GA had limited bias (0.11 weeks) and good precision compared to ultrasound GA, with 86% of predictions falling within ± 2 weeks.

In high-income countries, clinicians assess GA during the antenatal period using first or early second trimester ultrasound assessment of key fetal measurements. However, ultrasound requires relatively costly equipment with skilled operators, as well as first or early second trimester antenatal care in a clinic. In LICs, these requirements are infrequently met (13). In the absence of ultrasound assessments, GA is frequently based on last menstrual period. However, this estimate is inaccurate when compared to ultrasound due to varying menstrual cycle lengths in women, ovulation/conception timing, and recall error (14–16). Where antenatal assessment of GA is infrequent, postnatal GA scoring methods may be used, such as the Ballard and Dubowitz scores (17, 18). These methods are used in LICs, but also require skilled clinicians with ongoing training and may not perform as well in these settings. Low birth weight is frequently used as a proxy for prematurity due to the scarcity of better GA dating methods. However, most babies are not weighed at birth in LICs (2) and low birth weight is a poor proxy in populations with many growth-restricted neonates.

Smartphone ophthalmoscopy for ALCV is easy to conduct and rapid. It also has the advantage of being feasible in both healthy and sick neonates. ALCV imaging does not require special skills, as it is non-invasive. The PanOptic Ophthalmoscope allows a relatively wide view of ALCV compared to other direct ophthalmoscopes, avoiding the need for mydriatics, which are associated with apneas and bradycardia events in neonates (19, 20), and may increase risk of necrotizing enterocolitis (21, 22).

Original studies reported rapid regression of ALCV following delivery (5); however, later studies have reported a constant rate of decay (23, 24). We confirm that the rate of ALCV regression remains constant after delivery. This constant rate of ALCV decay could allow GA assessment at any time prior to 35 weeks when the vascularity has fully regressed. This is in contrast to other methods of postnatal GA assessment, which need to be performed shortly after delivery (17, 18). Most studies to date have not reported an effect of intrauterine growth on ALCV (25, 26). Future research is needed to elucidate the effect of medications and intrauterine stress from maternal and fetal conditions, including maternal hypertension (27) and intrauterine growth restriction (26), on the regression of ALCV.

Semiautomated image analysis allows the objective quantification of ALCV, including degree of branching, thickness, and density; thus, the estimation of GA may be more reliable than human readers. While the current iteration of software requires the transfer of the video to a computer for image analysis, future work entails developing a smartphone application, so that videos can be analyzed immediately after imaging, allowing rapid and reliable estimation of GA with minimal user input and labor.

There were several key challenges in the development of an automated image analysis application. The iPhone camera images are compressed and are of lower quality when compared to images captured by dedicated retinal ophthalmic imaging cameras. The image quality of iPhone 4s was noticeably inferior to iPhone 6s, forcing improvement of our software and methodology. Moreover, video images are affected by both the person performing the imaging and infant motion, which may induce defocusing artifacts. Another potential concern is inter-operator variability; however, we did not assess this in the current pilot study. In addition to light reflection, mucus can cause artifacts that resemble the target biomarkers. The image artifacts from mucus and surface-drying artifacts were countered by placing two drops of sterile saline on the eye prior to imaging. Corneal clouding can also impact the focusing ability of the camera and image quality.

While in the past few years several algorithms have been proposed to deal with artifacts in neonatal and adult retinal imaging applications (28–30), ALCV imaging is more challenging. Unlike most retinal imaging applications where the retina occupies the whole or most of the field-of-view, images of the cornea in our experiments occupied only a fraction of the total images acquired and were not in the same focal plane (depth) with the rest of the field-of-view. This is especially troublesome because it is not possible to estimate the quality of camera focus from any other region of the image but the corneal region. In addition, the lack of mydriatics may result in an obscured lens, without full visualization of the ALCV.

In contrast to retinal imaging, where some vascular features can be found even in the most severely diseased eyes, the lenses of neonates born >34 weeks gestation do not include vasculature. That is, unlike retinal imaging applications, it is not possible to directly use metrics based on the sharpness of vascular features to evaluate the quality of captured images in some cases. In fact, it is complex to distinguish an image of a cornea, which is truly devoid of vasculature, vs. one in which defocusing artifacts have concealed the appearance of vasculature. For this study, the stills were manually selected from videos or sets of images that were taken during imaging of our patients. Automated selection of images by the developed software was less reliable at the time of analysis, but this has since been improved with further development of the software. Future iterations of the software will utilize deep learning techniques, already proven impactful for many retinal image analyses (31, 32), to fully automate the process.

The goal of this pilot study was to determine the feasibility of combining smartphone ophthalmoscopy and semiautomated image analysis to quantify ALCV features and estimate GA among preterm neonates. While our sample size was small, the pilot provided key data for us to develop the software for semiautomated ALCV quantification and the predictive model for GA. We recently completed enrollment for a larger, multicenter study in three university hospitals in the United States to evaluate smartphone ophthalmoscope ALCV estimated gestational age. This expanded study will validate a more comprehensive neonatal assessment, including ALCV, metabolic markers, neonatal anthropometrics, and physical and neurological maturity scoring. This study had a larger sample size, with greater statistical power, and will allow for machine learning approaches to improve the prediction of gestational age.

In conclusion, this novel application of smartphone ophthalmoscopy and automated ALCV image analysis could provide a rapid, accurate, and non-invasive technology to objectively estimate postnatal GA in preterm neonates, especially in LICs where GA might not be known at birth.

## Data Availability

The raw data supporting the conclusions of this manuscript will be made available by the authors, without undue reservation, to any qualified researcher.

## Author Contributions

JG conceived and designed the study. MP and AB contributed to the design of the study. MP, AB, and JG enrolled patients and conducted data collection. DM and SF developed ALCVanalyzer software and conducted image analysis. BM performed the statistical analysis. CW and JG contributed to the analysis. MP wrote the first draft of the manuscript. MP, DM, SF, AB, and JG wrote sections of the manuscript. All authors contributed to manuscript revision, read, and approved the submitted version.

### Conflict of Interest Statement

The authors declare that the research was conducted in the absence of any commercial or financial relationships that could be construed as a potential conflict of interest.
